# Urine iodine concentration in hospitalised infants with thyroid dysfunction

**DOI:** 10.1186/s13044-025-00256-5

**Published:** 2025-08-07

**Authors:** Christy Hou, Michelle Jack, Annabelle Hobbs, Geoffrey Ambler, Yoon Hi Cho

**Affiliations:** 1 Faculty of Medicine and Health, The University of Sydney, Sydney, NSW Australia; 2 Faculty of Health Sciences and Medicine, Bond University, Queensland, Australia; 3 Institute of Endocrinology and Diabetes, The Children’s Hospital at Westmead, Sydney, NSW Australia

**Keywords:** Iodine, Infant, Urine iodine, Thyroid dysfunction, Hypothyroidism

## Abstract

**Background:**

Iodine is essential to thyroid hormone production, and both excess and deficiency can cause thyroid dysfunction in infants. While urinary iodine concentration (UIC) is used to assess population iodine status, there is no gold standard for determining iodine status in individual infants. Our study aimed to examine the clinical use of UIC in the investigation of thyroid dysfunction in hospitalised infants.

**Methods:**

We examined hospital records of infants (age < 24 months) admitted to The Children’s Hospital at Westmead who had UIC collected in the context of thyroid dysfunction between 2007–2009 and 2017–2021, two time periods separated by changes in public health measures for iodine nutrition and local clinical practice.

**Results:**

Of 152 infants, 13.8% had UIC in iodine deficient range (WHO population-based definition: UIC < 100 µg/L) and 53.9% in iodine excess range (UIC ≥ 300 µg/L). Highest quartile UIC (> 1432 µg/L) was significantly associated with pre-test clinician suspicion of iodine excess, identification of source of iodine exposure, higher percentage of premature babies, and those with cardiac anomalies or who required surgery. Median free thyroxine (fT4) level was significantly lower in the highest UIC quartile group compared to the lower three quartiles (9.4pmol/L [interquartile range 7.8-vs 13.7] vs. 12.7 pmol/L [10.3–15.6]; *p* = 0.004). While median TSH was elevated in all UIC quartiles in this group, there were no significant differences in the levels between the UIC quartile groups.

**Conclusions:**

Extremely high random UIC can be helpful to confirm clinical suspicion of iodine excess in hospital-based infants, taken in the context of thyroid dysfunction in critical illness. The degree of thyroid dysfunction associated with high UIC in this clinically complex and often premature patient population may be better measured by the fT4 level rather than the degree of TSH elevation.

## Background

Iodine is a trace element vital in the production of both thyroxine (fT4) and triiodothyronine (fT3) [1]. Both iodine deficiency and excess can cause transient hypothyroidism in infants [2], and iodine deficiency disorder in the neonatal period is globally a leading preventable cause of intellectual disability in children [1, 3, 4]. Mandatory iodine fortification of bread and the National Health and Medical Research Council (NHMRC) recommendation for iodine supplementation in all pregnant women were introduced in Australia in 2009 and 2010 respectively [5, 6]. However, despite fortification, pregnant women in Australia have a median urinary iodine concentration (UIC) of 116 µg/L [5], defined as iodine deficient (< 150 µg/L) by the World Health Organisation (WHO) [1]. The higher iodine target compared to the general population recognises that pregnancy is a particular time of risk for iodine deficiency as iodine requirements increase secondary to increased maternal thyroxine production requirements, iodine transfer to the fetal-placental unit and increased renal clearance of iodine [7, 8]. Maternal iodine deficiency can lead to neonatal iodine deficiency [3]. Conversely, iodine excess can cause thyroid dysfunction through the Wolff-Chaikoff effect, a phenomenon in which sudden iodine exposure leads to a transient paradoxical inhibition of iodide organification and thyroid hormone biosynthesis [9]. This phenomenon can be attributed to autoregulation by thyroid follicular cells as a protective mechanism against variation in dietary iodine intake. Preterm infants are at particular risk of prolonged hypothyroidism following exposure to excess iodine because the thyroid’s capacity to recover from the Wolff-Chaikoff effect is thought to mature after ~ 36 weeks of gestation [4]. Common causes of iodine excess in infants include exposure to topical iodine-containing antiseptics [10, 11], contrast media in surgery and high iodine content in breast milk due to excessive maternal dietary intake (e.g. excessive seaweed consumption) [11, 12].

Given its importance in thyroid metabolism, several methods have been used to assess iodine status in infants. UIC has been well studied as a measure for population screening for iodine deficiency in school age children [13, 14]. However, its use to determine iodine status of individual infants remains controversial. Consequently, the clinical use of UIC in the investigation of suspected iodine-related thyroid dysfunction remains a matter of contention, a gap in knowledge that our study endeavours to address.

The Children’s Hospital Westmead (CHW) is a tertiary and quaternary paediatric centre with a neonatal intensive care unit (NICU) and paediatric intensive care unit (PICU), receiving referrals for cardiac and other surgeries in infants, as well as those with complex congenital abnormalities requiring multidisciplinary care. Despite the controversy in the use of UIC in a clinical setting, it is routine practice at our hospital to request UIC as a part of the investigative work up for infants when abnormal iodine status is clinically considered be a contributing factor, or the aetiology of the hypothyroidism is unclear. In this study, our aim was to determine the clinical utility of UIC in hospitalised infants when ordered in the setting of thyroid dysfunction.

## Methods

Participants were identified from the hospital pathology records who had UIC measurements performed for clinical indications, collected between two pre-determined time periods, January 1st 2007 to December 31st 2009 and January 1st 2017 to December 31st 2021, and < 24 months of age at the time of the initial test. The two distinct time periods were chosen on either side of the years 2009–2010 when public health measures were introduced in Australia to prevent iodine deficiency in the general population and during pregnancy. Past Australian studies have also compared similar time periods, separated by the public health intervention, on newborn thyroid stimulating hormone (TSH) outcomes [15, 16]. The two time periods also captured the change in clinical practice, including increased use of UIC by clinicians at our centre and increased awareness of iodine toxicity [12]. UIC was measured in a single laboratory, minimising error due to inter-laboratory or inter-assay variation. Data were extracted from the hospital electronic medical records according to a predetermined data extraction sheet [17]. Details collected included demographics, birth history, infant feeding type, common neonatal complications (i.e. hypoglycaemia, jaundice) and NICU/PICU admissions as well as the neonatal clinical course and associated conditions (cardiac anomalies, surgery). Factors related to maternal history were extracted (including gestational diabetes mellitus and thyroid disease). Initial thyroid function tests (TFTs) included age when the test was ordered, TSH (reference range, RR, 0.4-5 mIU/L), fT4 (RR 13–30 pmol/L in the first 30 days; 10–20 pmol/L thereafter), fT3 (RR 3.5–6.5 pmol/L), reverse triiodothyronine (rT3, 140–540 pmol/L) and thyroglobulin levels, if measured, and the hospital department which ordered the TFTs. Data on urine iodine included indication for requesting the test, total UIC level (µg/L), clinical impression of the UIC result, whether there was any pre-test suspicion of neonatal iodine excess and any suspected sources of excess iodine exposure identified post-test. If the provisional diagnosis or clinical impression was not evident upon medical record review, it was categorised as unknown. Treatment course relating to levothyroxine was also extracted. Data from all patients were deidentified for analysis.

SPSS software v.25 was used to calculate median and interquartile range [IQR] for non-normally distributed data and mean and standard deviation (SD) for normally distributed data. Difference between categorical groups was assessed using the Chi-squared test, difference between medians between more than two comparison groups was compared using the Kruskal-Wallis H test, or between two groups using the Mann-Whitney U test, and difference between means between two groups using the T-test and between more than two groups using the one-way ANOVA. Fisher’s exact test or Fisher-Freeman-Halton test were used when appropriate (expected count < 5). Statistical significance was defined as *p* < 0.05.

Total UIC was categorised as deficient (< 100 µg/L), normal (100–299 µg/L) or excessive (*≥* 300 µg/L) according to the WHO definition of UIC population cut-offs in young children [18]. In view of its highly skewed distribution, UIC was also analysed as both a continuous variable and stratified into quartiles. Multivariate analysis of variance was performed to examine the multivariable relationship between UIC quartile and initial thyroid function: TSH, fT4 and fT3, adjusted for confounders and interaction terms.

## Results

UIC results were available for 155 infants over the periods of 2007–2009 and 2017–2021, with three patients excluded after review of their medical records because there was no clinical context for the UIC order and appeared to have been ordered in error. The final sample size was 152 patients, with 25 patients in the 2007–2009 group and 127 patients in the 2017–2021 group.

### Population characteristics

Baseline clinical and biochemical characteristics for the study population, stratified by the time period, are available in Table 1. Compared to the New South Wales (NSW) population in 2020, our study cohort had higher percentages of infants who were premature, defined as a gestational age of less than 37 weeks (7.3% in NSW vs. 38.7%), born via Caesarean section (36.7% in NSW vs. 47.4%) and with a low birthweight, defined as less than 2500 g (6.6% in NSW vs. 36.2%) [19]. The recent cohort of infants (time period 2017–2021), compared to the first cohort (time period 2007–2009), had a higher percentage who were premature (42.9% vs. 16.7%; *p* = 0.017), higher percentage who required emergency Caesarean section (32.3% vs. 4.5%) and had lower median birthweight (3190 g [2580–3500] vs. 2689 g [2059–3250]; *p* = 0.017). The recent cohort of infants were more clinically complex with greater neonatal comorbidities and cardiac anomalies (Table 1).

**Table 1:** Overall baseline clinical and biochemical characteristics and stratified by time period

		Frequency (percentage)			*p*-value
		Whole group (*n* = 152)	2007–2009 (*n* = 25)	2017–2021 (*n* = 127)	
**Demographics**	Sex (male)	93/152 (61.2%)	15/25 (60.0%)	78/127 (61.4%)	0.894
	Premature	58/150 (38.7%)	4/24 (16.7%)	54/126 (42.9%)	0.017^a^
	Gestational age (weeks)	37.6 [35.1–39.3]	39.0 [36.5–40.3]	37.0 [35.0–39.0]	0.004
	Multiple birth	11/141 (7.8%)	0/21 (0.0%)	11/120 (9.2%)	0.081^a^
**Birth history**	Normal vaginal delivery	62/133 (46.6%)	16/22 (72.7%)	46/111 (41.4%)	0.012^a^
	Emergency Caesarean section	43/133 (32.3%)	1/22 (4.5%)	42/111(37.8%)	
	Elective Caesarean section	20/133 (15.0%)	4/22 (18.2%)	16/111 (14.4%)	
	Assisted delivery	8/133 (6.0%)	1/22 (4.5%)	7/111 (6.3%)	
	Birthweight (grams)	2800 [2180-3310]	3190 [2580-3500]	2689 [2059-3250]	0.017
	Low birth weight	51/141 (36.2%)	4/23 (17.4%)	47/118 (39.8%)	0.098^a^
	Very low birth weight	19/141(13.5%)	1/23 (4.3%)	18/118 (15.3%)	0.457^a^
**Neonatal comorbidities**	Neonatal hypoglycaemia	34/117 (29.1%)	0/14 (0.0%)	34/103 (33.0%)	0.002
	Jaundice	70/125 (56.0%)	10/16 (62.5%)	60/109 (55.0%)	0.042^a^
	NEC	9/129 (7.0%)	2/17 (11.8%)	7/112 (6.3%)	0.021^a^
	HIE	2/129 (1.6%)	0/17 (0.0%)	2/112 (1.8%)	0.062^a^
	Respiratory distress	81/126 (64.3%)	8/17 (47.1%)	73/109 (67.0%)	0.031^a^
	SCN admission	19/124 (15.3%)	2/17 (11.8%)	17/107 (15.9%)	0.170^a^
	NICU admission	98/124 (79.0%)	10/17 (58.8%)	88/107 (82.2%)	0.019^a^
**Maternal factors**	Maternal antenatal steroids	30/125 (24.0%)	1/16 (6.3%)	29/109 (26.6%)	0.011^a^
	Maternal gestational diabetes	25/135 (18.5%)	1/20 (5.0%)	24/115 (20.9%)	0.072^a^
	Maternal thyroid disease	24/144 (16.7%)	1/21 (4.8%)	23/123 (18.7%)	0.017^a^
**Medical history**	Trisomy 21	17/152 (11.2%)	2/25 (8.0%)	15/127 (11.8%)	0.782^a^
	Cardiac anomaly	93/152 (61.2%)	8/25 (32.0%)	85/127 (66.9%)	0.001
	Medications associated with thyroid dysfunction	39/152 (25.7%)	5/25 (20.0%)	34/127 (26.8%)	0.738^a^
	Post-surgery	98/152 (64.5%)	10/25 (40.0%)	88/127 (69.3%)	0.006

Denominators vary due to missing data. Results presented as median [IQR]

Abbreviations: NEC = Necrotizing enterocolitis, HIE = Hypoxic-Ischemic Encephalopathy, SCN = Special Care Nursery, NICU = Neonatal Intensive Care Unit

^a^*p*-value calculated using Fisher’s exact test or Fisher-Freeman-Halton test due to expected count < 5

### Thyroid assessment

All infants who had UIC test ordered also had thyroid dysfunction documented. TFTs were ordered by staff from the NICU in 53/152 (34.9%) of infants, PICU in 38/152 (25.0%), general paediatric teams in 24/152 (15.8%) and the endocrinology team in 17/152 (11.2%). The median age of the infants in this study cohort at the time of the initial TFT was 29.5 days [IQR 13.3–67.8]. Initial pre-treatment thyroid function of the total group showed a mildly elevated median TSH 8.52mIU/L [IQR 6.11–17.55], low normal range fT4 12.3pmol/L [IQR 9.4–15.2] and normal fT3 4.2pmol/L [IQR 2.9–5.6]. Median reverse T3 was 1345.5pmol/L [683.5-2063.5]. Repeat TFTs were ordered in 145/152 (95.4%) and found to be abnormal on serial testing in 34/145 (23.4%), although some of these results would have been taken on treatment. Only 5/152 (3.3%) of patients in this study had serum thyroglobulin levels measured. Thyroid ultrasound was performed in 71/152 (46.7%) of infants and categorised into the following findings: normal (*n* = 59, 83.1%), enlarged thyroid gland (*n* = 4, 5.6%), dysgenesis (*n* = 5, 7.0%) and nodules (*n* = 3, 4.2%). Nuclear medicine thyroid scan was performed in 62/152 (40.8%) of infants and categorised into the following findings: normal uptake (*n* = 16, 25.8%), reduced uptake (*n* = 21, 33.9%), increased uptake (*n* = 18, 29.0%) and no uptake (*n* = 7, 11.3%).

### UIC

The median age at UIC collection was 37.0 days [22.0-87.5], with median UIC 353.0 µg/L [145.0-1432.8]. The median interval between TFT measurement and UIC collection was five days [1.0–14.0]. Repeat UIC was performed in 29/152 infants (19.1%). According to the WHO classification, UIC results were in normal range in 49/152 infants (32.2%), iodine deficient range in 21/152 (13.8%) and excessive range in 82/152 (53.9%). There was a pre-test suspicion of iodine excess in 45/152 of infants (29.6%). After the UIC results, sources of excess iodine exposure were identified in 60/152 (39.5%).

Clinical and biochemical characteristics stratified by total UIC quartiles are described in Table 2. While median TSH was mildly elevated in all UIC quartile groups, there was no significant difference between the UIC quartile groups (Table 2). Median fT4 was significantly lower in infants with the highest UIC quartile compared to the lower three UIC quartiles (9.4pmol/L [7.8-vs 13.7] vs. 12.7pmol/L [10.3–15.6]; *p* = 0.004). However, there was no significant difference in median fT4 levels in infants with UIC in the upper two quartiles vs. lower two quartiles, nor infants with UIC in the upper three quartiles vs. lowest quartile. Similarly, median fT3 was significantly lower in the highest UIC quartile (4.7 vs. 5.75pmol/L; *p* = 0.006).

**Table 2:** Clinical and biochemical characteristics stratified by total UIC quartiles

Clinical/biochemical characteristics	UIC Quartile 1 (*n* = 39)	UIC Quartile 2 (*n* = 37)	UIC Quartile 3 (*n* = 38)	UIC Quartile 4 (*n* = 38)	*p*-value
UIC range (µg/L)	< 146	146–353	354–1432	> 1432	
Gestational age (weeks)	37.1 [35.2–39.6]	39.0 [35.5–40.0]	36.7 [35.0-38.7]	37.7 [36.1–39.5]	0.075
Birthweight (grams)	2865 [1988–3340]	2960.0 [2500–3500]	2490.0 [1901–3130]	2920 [2223–3330]	0.126
Age at referral (days)	32 [20–169]	49 [28–99]	35 [22–109]	26.0 [15–52]	0.057
Age when TFTs taken (days)	37 [14–209]	38 [21–64]	32 [13–85]	19 [11–47]	0.053
TSH (mIU/L)	7.72 [5.18–12.6]	7.40 [4.40-17.71]	9.25 [6.72–21.10]	9.61 [6.30–21.60]	0.354
fT4 (pmol/L)^a^	12.4 [10.0-15.4]	13.5 [11.2–17.1]	12.5 [9.7–15.6]	9.4 [7.8–13.7]	0.005
fT3 (pmol/L)^a^	4.3 [3.8–5.4]	5.6 [3.1–6.7]	4.3 [3.2–5.5]	2.9 [2.1–4.7]	0.013
	*Frequency (percentage)*				
Premature (< 37 weeks)	15/39 (38.5%)	9/35 (25.7%)	20/38 (52.6%)	14/38 (36.8%)	0.082^a^
Multiple birth	3/36 (8.3%)	2/30 (6.7%)	5/36 (13.9%)	1/37 (2.7%)	0.063^a^
NICU admission	26/34 (76.5%)	16/23 (69.6%)	29/35 (82.9%)	27/32 (84.4%)	0.020
Trisomy 21	6/39 (15.4%)	3/37 (8.1%)	8/38 (21.1%)	0/38 (0.0%)	0.009^a^
Cardiac anomaly	18/39 (46.2%)	17/37 (45.9%)	29/38 (76.3%)	29/38 (76.3%)	0.002
Medications associated with thyroid dysfunction	9/39 (23.1%)	3/37 (8.1%)	10/38 (26.3%)	17/38 (44.7%)	0.007^a^
Pre-test suspicion of iodine excess	5/39 (12.8%)	7/37 (18.9%)	15/38 (39.5%)	18/38 (47.4%)	0.002
Post-surgery	23/39 (59.0%)	14/37 (37.8%)	26/38 (68.4%)	35/38 (92.1%)	< 0.001
Source of iodine excess identified	4/39 (10.3%)	9/37 (24.3%)	19/38 (50.0%)	28/38 (73.7%)	< 0.001
Treatment started	20/39 (51.3%)	23/37 (62.2%)	28/38 (73.7%)	26/38 (68.4%)	0.201
Repeat UIC ordered	3/39 (7.7%)	4/37 (10.8%)	8/38 (21.1%)	14/38 (36.8%)	0.005

Denominators vary due to missing data. Results presented as median [IQR]

^a^*p*-value calculated using Fisher-Freeman-Halton Exact Test due to expected count < 5

In univariate analyses, highest UIC quartile (compared to lower three quartiles) was significantly associated with lower fT4 (b=-2.74 [-4.59 - -0.89]; *p* = 0.004; R^2^ = 0.05), lower birthweight (ß = 0.001 [0.000-0.002], R^2^ = 5.4%, *p* = 0.006) and lower gestational age (ß = 0.24 [0.012–0.45], R^2^ = 3.2%, *p* = 0.036), but not with the age at referral. In multivariate analysis of variance, highest UIC quartile and birthweight were independently associated with lower fT4 as a continuous outcome and together accounted for 9.6% of the variability in fT4 (adjusted R^2^ = 9.6%; *p* < 0.0005).

During the first time period 2007–2009, 5/25 (20.0%) infants were classified as having iodine deficiency, compared to 16/127 (12.6%) during the second time period 2017–2021. A significantly higher percentage of infants were classified as having iodine excess in the second time period 2017–2021 compared to the first time period 2007–2009 (43% vs. 16%; *p* = 0.01). There was no association between time period as an explanatory variable and thyroid function outcomes.

### Clinical impression and diagnoses based on UIC results

Final clinical impression documented for this cohort included excess iodine exposure in 38.8% (36/152 considered to have a combination of iodine toxicity and sick euthyroid syndrome, 14/152 confirmed to have infant iodine exposure and 9/152 with a maternal history of iodine excess) while a further three patients were categorised by the clinician as iatrogenic (3/152, 2.0%). UIC levels were in range of iodine deficiency in 13.8% of infants (21/152). Unrelated to iodine status, other causes for the thyroid dysfunction in this hospitalised cohort were sick euthyroid syndrome (30/152, 19.7%), dysgenesis (6/152, 3.9%), dyshormonogenesis (12/152, 7.9%), central hypothyroidism (4/152, 2.6%) and maternal thyroid autoantibodies (1/152, 0.7%). Aetiology in the remainder (34/152, 22.4%) were unknown or patients were lost to follow-up. Levothyroxine treatment was commenced in 57/82 (69.5%) of infants with an elevated UIC.

### Extremes of TSH and UIC

TSH > 50mIU/L was detected in 13 infants and UIC > 20,000 µg/L in 12 infants, whom we further describe in Tables 3 and 4 respectively, categorised by suspected sources of iodine exposure. Clinical diagnosis of iodine excess was made in 54% (7/13) of infants with extremely high TSH > 50mIU/L and 92% (11/12) of infants with an extremely high UIC > 20,000 µg/L. Not all cases with severe hypothyroidism (TSH > 50 mIU/L) due to suspected iodine excess demonstrated elevated UIC (cases 4 and 9 had UIC < 300 µg/L). Even in the context of extremely elevated UIC, some infants had a normal TSH level (although UIC and TSH were not always paired samples), while all except one infant (term gestation) had fT4 under the reference range. Nuclear medicine thyroid scan findings were variable.

**Table 3:** Cases of infants with TSH > 50 mIU/L (*n* = 13)

Case	Age (days)	Gestational age (weeks)	TSH (mIU/L)	fT4 (pmol/L)	fT3 (pmol/L)	Thyroid scan	Thyroid Ultrasound	UIC (µg/L)	Clinical risk of iodine excess	Clinician diagnosis
1	86	36.0	231.65	5.2	2.9	Not performed	Normal	1564	Cardiac surgery	Dysgenesis
2	20	38.4	70.43	9.4	6.0	Normal	Normal	817	Cardiac surgery	Iodine toxicity/sick euthyroid
3	61	38.6	328.57	9.3	6.8	Not performed	Not performed	18,860	Cardiac surgery	Iodine toxicity/sick euthyroid
4	25	39.6	75.57	6.4	2.9	Increased uptake	Normal	153	Cardiac surgery	Iodine toxicity/sick euthyroid
5	14	38.7	61.45	8.1	3.8	Increased uptake	Normal	4663	Cardiac surgery, LSCS	Neonatal iodine exposure
6	26	36.0	53.99	11.5	2.8	Increased uptake	Normal	97,825	Contrast study	Neonatal iodine exposure
7	1	33.0	156.04	16.1	-	Not performed	Not performed	41	LSCS	Iatrogenic (trans-placental transfer of PTU)
8	38	27.0	100.00	5.8	-	Normal	Normal	307	Maternal ingestion seaweed soup	Maternal iodine excess
9	38	27.0	66.47	7.6	-	Normal	Normal	72	Maternal ingestion seaweed soup	Maternal iodine excess
10	7	33.0	159.66	3.2	4.4	Not performed	Not performed	116	None	Iatrogenic (trans-placental transfer of PTU)
11	560	39.3	53.54	8.3	6.6	Increased uptake	Normal	597	None	Dyshormonogenesis
12	9	40.1	76.50	9.1	5.0	Increased uptake	Enlarged	195	None	Dyshormonogenesis
13	28	41.0	98.24	11.0	2.6	No uptake	Absent	269	None	Dysgenesis

Abbreviations: LSCS = Lower Segment Caesarean Section, PTU = propylthiouracil

**Table 4:** Cases of infants with UIC > 20,000 µg/L (*n* = 12)

Case	Age at referral (days)	Gestational age (weeks)	UIC (µg/L)	Clinical risk of iodine excess	TSH (mIU/L)	fT4 (pmol/L)	fT3 (pmol/L)	Thyroid scan	Thyroid Ultrasound	Clinician diagnosis
1	37	39.0	553,513	Cardiac surgery	9.47	8.8	2.2	Not performed	Not performed	Iodine toxicity/sick euthyroid
2	31	40.0	24,366	Cardiac surgery	8.01	8.7	-	Increased uptake	Normal	Iodine toxicity/sick euthyroid
3	12	41.0	37,922	Cardiac surgery	0.27	8.9	1.5	Not performed	Normal	Iodine toxicity/sick euthyroid
4	29	35.0	63,804	Cardiac surgery	21.56	9.4	-	Not performed	Not performed	Neonatal iodine exposure
5	8	35.9	22,088	Cardiac surgery, LSCS	6.52	5.4	-	Not performed	Not performed	Iodine toxicity/sick euthyroid
6	22	40.0	53,973	Cardiac surgery, LSCS	6.57	15.5	1.7	Not performed	Normal	Iodine toxicity/sick euthyroid
7	26	36.0	97,825	Contrast study	53.99	11.5	2.8	Increased uptake	Normal	Neonatal iodine exposure
8	77	38.6	53,537	LSCS	10.78	8.2	-	Not performed	Not performed	Sick euthyroid
9	20	25.7	23,525	LSCS, maternal Graves’ disease - on carbimazole	21.16	7.6	2.3	Not performed	Not performed	Neonatal iodine exposure
10	37	39.9	20,148	Maternal consumption of seaweed soup, LSCS	33.83	9.2	6.1	Normal	Not performed	Maternal iodine excess
11	428	35.0	32,995	Surgery	6.22	6.2	1.5	Not performed	Not performed	Iodine toxicity/sick euthyroid
12	354	37.7	31,956	Surgery	0.33	8.0	2.8	Not performed	Not performed	Iodine toxicity/sick euthyroid

Abbreviations: LSCS = Lower Segment Caesarean Section

## Discussion

The utility of UIC in population screening for iodine deficiency is well-established [1]. However, controversy remains regarding its application in assessing individual infants with thyroid hormone dysfunction. Our study suggests that UIC has a diagnostic role in confirming extreme iodine excess where there is clinical suspicion, particularly in unwell infants with a complex medical background and clinical risk of iodine excess.

In our single centre study of hospitalised infants, extremely high UIC was clinically useful in confirming clinician suspicion of iodine exposure or, upon receiving an unexpectedly high result during screening for causes of hypothyroidism in this clinical context, confirming the presence of iodine exposure. Our study cohort represented infants with clinical comorbidities: who were unwell, preterm, had lower birthweight or required surgery. Consistent with our findings, previous studies have found a strong association between elevated UIC and iodine exposure in preterm and term infants [20, 21]. Despite long-standing awareness since the 1980s that iodine toxicity secondary to contrast media and topical antiseptics can cause transient neonatal hypothyroidism [10, 22–24], iatrogenic iodine exposure remains an issue in current neonatal and intensive care units; the source of iodine exposure was identified in 39.5% of the high risk infants in this study. Notably, this is a highly selected population different to well infants in the community, and not representative of all preterm or unwell infants who may also be at risk of iodine deficiency secondary to maternal deficiency or low post-natal intake [3, 25, 26].

There was a significant relationship between the highest quartile of UIC and hypothyroidism, as defined by lower median fT4 and fT3, but not with TSH. Similarly, other studies have reported an association of UIC with lower fT4, but not TSH [21]. This may be explained by co-existence of the sick euthyroid syndrome in this unwell cohort of infants, resulting in variable TSH levels. Furthermore, it is well recognised that premature infants are unable to mount a rapid or sufficient TSH response due to immaturity of the hypothalamic-pituitary-thyroid axis [10, 27]. The lack of association between UIC and TSH may also be explained by the timing of TFTs in relation to UIC measurements. Other studies have found an association between UIC and TSH only when TFTs were measured more than seven days after UIC, or within 14 days of iodine exposure [10]. In our study population, UIC was generally measured after the detection of thyroid dysfunction, at a median interval of five days post-TFT measurement, although the exact timing of the preceding iodine exposure was variable.

The prevalence of iodine excess was significantly higher during the second time period (2017–2021) compared to the first (2007–2009), the second time period also having higher percentage of infants with prematurity and cardiac anomalies, requiring surgery and NICU admission. The latter time period therefore consisted of a more unwell infant population who were both more likely to have been exposed to topical iodine within a NICU and surgical setting and, being preterm and of low birthweight, more susceptible to the effects of excess iodine exposure. Increased UIC testing numbers in the recent time period reflects a change in local practice to include UIC measurement in the work-up of infants with clinical suspicion of iodine excess and coincides with increased awareness of the importance of iodine nutrition both in terms of public measures to prevent iodine deficiency and clinical experience of iodine excess [12].

In our study, we also had 14% of infants with UIC in the population definition of iodine deficiency, although the implication of a single UIC value for an individual is inconclusive. Nevertheless, the reduction in the percentage of infants with UIC in deficient range in the second time period compared to the first suggests that the introduction of public health measures have lowered, but not eliminated, the prevalence of iodine deficiency even in this specific cohort of unwell hospitalised infants. It must be recognised that preterm infants on parental nutrition are known to be at risk of iodine deficiency [25]. While clinicians may record suspicion of excess iodine exposure within the hospital setting, the underlying risk factors for iodine deficiency are rarely documented in the medical records. It would also be important to examine specifically for the risk factors for iodine deficiency in this high-risk population of hospitalised infants.

UIC can vary considerably with the iodine content of the individual’s diet, at different times of day and between individuals [28]. Infant iodine stores at birth and postnatally will be affected by maternal iodine status during pregnancy and lactation, particularly for neonates consuming breastmilk [29]. Maternal dietary iodine intake was identified as excessive in a small number of cases however it was generally poorly documented. Maternal dietary history is often overlooked and can be difficult to quantify; however, it is an important component of history taking when investigating iodine excess or deficiency in infants.

There is lack of consensus on normal ranges when UIC is used as a measure of iodine status in individual infants with abnormal thyroid function. WHO population UIC thresholds were designed to detect iodine deficiency in school age children and pregnant women [1], but their use on an individual basis is controversial; there is paucity of literature on even population UIC levels in infants [30]. In line with the threshold for school-age children, infant UIC *≥* 100ug/L is defined by the WHO as sufficient iodine status for children < 2 years [18], and also correlates with dietary iodine intake in this age group [31, 32]. The UIC levels in this study were highly skewed with several extremely high values, in excess of the variations which typically reflect iodine nutrition status of healthy infants in population-based studies. Stratifying UIC into quartiles allowed for categorisation into low, normal, mildly elevated and extremely elevated UIC levels. UIC in the second highest quartiles (similar UIC threshold to ‘excessive’ population iodine) was not significantly associated with hypothyroidism. We postulate that at the extremely high end of UIC, the intra-individual variability will be negligible compared to the magnitude of UIC elevation. Thus, a higher UIC threshold of *≥* 1,500 µg/L may be more appropriate in confirming iodine toxicity as the cause of hypothyroidism in this clinical scenario. A similar threshold was found in a systematic review of UIC in preterm infants in which those exposed to topical iodine had a higher UIC range 1,100 − 18,900 µg/L compared to those not exposed [21]. In our study, the finding of 11/12 (91.7%) of infants with elevated UIC > 20,000 µg/L being confirmed to have been exposed to excess iodine provides further evidence that very high UIC values can reliably support a clinical diagnosis of iodine excess as the final aetiology for thyroid dysfunction. It is possible that some infants may also have underlying genetic dyshormonogenesis, placing them at higher risk of thyroid dysfunction due to abnormalities in iodine status. Genetic testing may further elucidate the presence of underlying dyshormonogenesis, although this is not routinely done in an acute setting. The apparent variability in thyroid function response to the degree of elevation in UIC may have also resulted from a mismatch between the time course of thyroid dysfunction after iodine exposure, and the timing of UIC measurements. Serial paired measurements would help to further investigate the relationship between elevation in UIC and transient thyroid dysfunction in this patient group, taking into account other confounders including prematurity, sick euthyroid syndrome and nutritional iodine intake.

At our centre, UIC results typically have a one week turnaround time and may not be available in the acute setting. These results nevertheless have a role in confirming clinical suspicion of iodine excess or identify the source of iodine excess in hospitalised infants, particularly during the course of their often prolonged admission. In addition, UIC results may retrospectively help to confirm a transient cause of hypothyroidism, and if thyroxine replacement was commenced, allow earlier trial off therapy.

Limitations include the retrospective nature of the study, resulting in missing data and lack of follow up to final diagnoses in those who were discharged or had their care transferred locally. Our study has limited generalisability for UIC measurement to assess individual iodine status in healthy infants in the community, as this study only included infants admitted at a tertiary paediatric centre who had UIC requested for thyroid dysfunction, mostly in the intensive care setting. Furthermore, as this was a highly selected cohort who already had thyroid dysfunction, the results do not provide a general prevalence or risk of hypothyroidism in the context of iodine excess in hospitalised infants.

There remains no gold standard investigation of infant iodine status on an individual level. Aside from spot UIC, which this study investigated, there have been studies investigating serum thyroglobulin, 24-hour UIC, serum iodine and salivary iodine as potential measures of iodine status. Further studies are warranted to adequately describe the iodine nutrition status and risks of both excess and deficiency in this vulnerable patient population.

## Conclusions

We propose that an extremely high random UIC *≥* 1,500 µg/L on an individual level may assist clinicians in confirming clinical suspicion or identifying previously unsuspected source of iodine excess in hospitalised infants. However, there is insufficient evidence of the strength of the relationship between UIC and thyroid dysfunction, with multiple clinical variables that likely impact thyroid function in this highly specific group of unwell and preterm infants; and a normal UIC level does not exclude iodine excess. Prospective longitudinal studies comparing serial UIC in infants with both normal and abnormal thyroid function, and exploration of alternative clinical measures of iodine status, will help elucidate the relationship between iodine status and thyroid function in this clinically complex patient population.

## Data Availability

The datasets generated and/or analysed during the current study are not publicly available due to institutional ethics requirements to protect the privacy of the participants.

## Abbreviations

NHRMC: National Health and Medical Research Council
UIC: Urine iodine concentration
WHO: World Health Organisation
CHW: The Children’s Hospital at Westmead
NICU: Neonatal intensive care unit
PICU: Paediatric intensive care unit
TFT: Thyroid function test
TSH: Thyroid stimulating hormone
fT4: Free thyroxine
fT3: Free triiodothyronine
rT3: Reverse triiodothyronine
IQR: Interquartile range
SD: Standard deviation
NSW: New South Wales

## References

[1] WHO. Assessment of iodine deficiency disorders and monitoring their elimination2007; (3rd Edition). Available from: https://www.who.int/publications/i/item/9789241595827

[2] Sohn SY, Inoue K, Rhee CM, Leung AM. Risks of iodine excess. Endocr Rev. 2024;45(6):858–79. 38870258 10.1210/endrev/bnae019

[3] Rastogi MV, LaFranchi SH. Congenital hypothyroidism. Orphanet J Rare Dis. 2010;5:17. 20537182 10.1186/1750-1172-5-17 PMC2903524

[4] Wassner AJ. Congenital hypothyroidism. Clin Perinatol. 2018;45(1):1–18. 29405999 10.1016/j.clp.2017.10.004

[5] AIHW. Monitoring the health impacts of mandatory folic acid and iodine fortification Canberra: Australian Institute of Health and Welfare. 2016 [Cat. no. PHE 208:[Available from: https://www.aihw.gov.au/getmedia/6bfafa4a-2255-4f04-8955-7496c9e5b2c1/19192.pdf?v=20230605183245&inline=true

[6] NHMRC. Iodine supplementation for pregnant and breastfeeding women 2010 [Available from: https://www.nhmrc.gov.au/about-us/publications/iodine-supplementation-pregnant-and-breastfeeding-women#toc__2

[7] Glinoer D. The regulation of thyroid function during normal pregnancy: importance of the iodine nutrition status. Best Pract Res Clin Endocrinol Metab. 2004;18(2):133–52. 15157832 10.1016/j.beem.2004.03.001

[8] Perez-Lopez FR. Iodine and thyroid hormones during pregnancy and postpartum. Gynecol Endocrinol. 2007;23(7):414–28. 17701774 10.1080/09513590701464092

[9] Wolff J, Chaikoff IL. Plasma inorganic iodide as a homeostatic regulator of thyroid function. J Biol Chem. 1948;174(2):555–64. 18865621

[10] Smerdely P, Lim A, Boyages SC, Waite K, Wu D, Roberts V, et al. Topical iodine-containing antiseptics and neonatal hypothyroidism in very-low-birthweight infants. Lancet. 1989;2(8664):661–4. 2570908 10.1016/s0140-6736(89)90903-3

[11] Connelly KJ, Boston BA, Pearce EN, Sesser D, Snyder D, Braverman LE, et al. Congenital hypothyroidism caused by excess prenatal maternal iodine ingestion. J Pediatr. 2012;161(4):760–2. 22841183 10.1016/j.jpeds.2012.05.057 PMC4354797

[12] Crawford BA, Cowell CT, Emder PJ, Learoyd DL, Chua EL, Sinn J, et al. Iodine toxicity from soy milk and seaweed ingestion is associated with serious thyroid dysfunction. Med J Aust. 2010;193(7):413–5. 20919974 10.5694/j.1326-5377.2010.tb03972.x

[13] Wainwright P, Cook P. The assessment of iodine status - populations, individuals and limitations. Ann Clin Biochem. 2019;56(1):7–14. 29703103 10.1177/0004563218774816

[14] Zimmermann MB. Iodine deficiency. Endocr Rev. 2009;30(4):376–408. 19460960 10.1210/er.2009-0011

[15] Yu A, Alder N, Lain SJ, Wiley V, Nassar N, Jack M. Outcomes of Lowered newborn screening thresholds for congenital hypothyroidism. J Paediatr Child Health. 2023;59(8):955–61. 37184332 10.1111/jpc.16425

[16] Lain SJ, Roberts CL, Wilcken B, Wiley V, Jack MM, Nassar N. Using record linkage to investigate perinatal factors and neonatal thyroid-stimulating hormone. J Paediatr Child Health. 2015;51(6):620–5. 25425135 10.1111/jpc.12783

[17] Hobbs A, Jack M, Benitez-Aguirre P, Srinivasan S, Wotton T, Cho YH. Challenges in management of abnormal thyroid function tests in unwell infants: A tertiary centre real-world experience. J Paediatr Child Health. 2025;61(2):185–90. 39632356 10.1111/jpc.16733

[18] WHO. Urinary iodine concentrations for determining iodine status in populations 2013 [WHO/NMH/NHD/EPG/13.1]. Available from: https://iris.who.int/bitstream/handle/10665/85972/WHO_NMH_NHD_EPG_13.1_eng.pdf?sequence=1

[19] New South Wales Mothers and Babies. 2020 Sydney2021 [Available from: https://www.health.nsw.gov.au/hsnsw/Publications/mothers-and-babies-2020.pdf

[20] Bona G, Chiorboli E, Rapa A, Weber G, Vigone MC, Chiumello G. Measurement of urinary iodine excretion to reveal iodine excess in neonatal transient hypothyroidism. J Pediatr Endocrinol Metab. 1998;11(6):739–43. 9829229 10.1515/jpem.1998.11.6.739

[21] Aitken J, Williams FL. A systematic review of thyroid dysfunction in preterm neonates exposed to topical iodine. Arch Dis Child Fetal Neonatal Ed. 2014;99(1):F21–8. 24105624 10.1136/archdischild-2013-303799

[22] l’Allemand D, Gruters A, Beyer P, Weber B. Iodine in contrast agents and skin disinfectants is the major cause for hypothyroidism in premature infants during intensive care. Horm Res. 1987;28(1):42–9. 3447940 10.1159/000180924

[23] Harada SI, Arai N, Honma J, Matsuura H, Fujieda N. Influence of iodine excess due to iodine-containing antiseptics on neonatal screening for congenital hypothyroidism in Hokkaido Prefecture. Japan Screen. 1994;131(3):434–9.

[24] Ryan CA, Hallgren RA, Finer NN. The use of Povidone iodine in neonatal bowel surgery. J Pediatr Surg. 1987;22(4):317–9. 3572688 10.1016/s0022-3468(87)80232-4

[25] Belfort MB, Pearce EN, Braverman LE, He X, Brown RS. Low iodine content in the diets of hospitalized preterm infants. J Clin Endocrinol Metab. 2012;97(4):E632–6. 22337912 10.1210/jc.2011-3369 PMC3319182

[26] Nazeri P, Akbarzadeh M, Pearce EN, Hedayati M, Dalili H. Iodine status of preterm infants born in an area of iodine sufficiency: are they at risk of iodine deficiency?? Endocr Pract. 2022;28(9):835–41. 35671879 10.1016/j.eprac.2022.05.010

[27] Williams FL, Mires GJ, Barnett C, Ogston SA, van Toor H, Visser TJ, et al. Transient hypothyroxinemia in preterm infants: the role of cord Sera thyroid hormone levels adjusted for prenatal and intrapartum factors. J Clin Endocrinol Metab. 2005;90(8):4599–606. 15886240 10.1210/jc.2005-0214

[28] Rasmussen LB, Ovesen L, Christiansen E. Day-to-day and within-day variation in urinary iodine excretion. Eur J Clin Nutr. 1999;53(5):401–7. 10369497 10.1038/sj.ejcn.1600762

[29] Andersson M, Braegger CP. The role of iodine for thyroid function in lactating women and infants. Endocr Rev. 2022;43(3):469–506. 35552681 10.1210/endrev/bnab029 PMC9113141

[30] Wallborn T, Vogel M, Kneuer A, Thamm M, Dittrich K, Kiess W, et al. Spot urine iodine levels below the WHO recommendation are not related to impaired thyroid function in healthy children and adolescents. Eur J Nutr. 2021;60(1):493–502. 32390124 10.1007/s00394-020-02268-3 PMC7867514

[31] Fallah R, Du L, Braverman LE, He X, Segura-Harrison M, Yeh MW, et al. Iodine nutrition in weaning infants in the united States. Thyroid. 2019;29(4):573–6. 30827204 10.1089/thy.2018.0102 PMC6457884

[32] Naess S, Aakre I, Strand TA, Dahl L, Kjellevold M, Stokland AM, et al. Infant iodine status and associations with maternal iodine nutrition, breast-feeding status and thyroid function. Br J Nutr. 2023;129(5):854–63. 35535981 10.1017/S0007114522001465 PMC9975782

